# Supplementary material for: Deep learning based ECG segmentation for delineation of diverse arrhythmias
Source: PLoS One. 2024 Jun 13;19(6):e0303178. doi: 10.1371/journal.pone.0303178 (PMC11175442; doi:10.1371/journal.pone.0303178)
Supplement: S1 Appendix — (PDF) [file pone.0303178.s001.pdf]

Supporting information

Appendix A. As a quality control measure of the waveform boundary annotations for the internal dataset, we include in Table 1 the standard deviation of the difference between lead I and lead II annotation. Smaller values means that the annotated points are closer. In particular, we see that the annotation quality of the T waves is less consistent than that of the QRS complexes.

	P onset	P offset	QRS onset	QRS offset	T onset	T offset
Std (ms)	7.3	6.7	2.4	3.2	7.8	7.8

Table 1. Standard deviation of difference in lead I and lead II annotation.
